# Comparison of Two Surgical Approaches for Coronary Artery Bypass of Left Anterior Descending Artery

**DOI:** 10.3390/jcm13113158

**Published:** 2024-05-28

**Authors:** Saad Salamate, Farhad Bakhtiary, Ali Bayram, Sami Sirat, Mirko Doss, Veaceslav Ciobanu, Nadejda Monsefi, Ali El-Sayed Ahmad

**Affiliations:** 1 Department of Cardiac Surgery, University Hospital Bonn, 53127 Bonn, Germany; farhad.bakhtiary@ukbonn.de (F.B.); veaceslav.ciobanu@ukbonn.de (V.C.); nadejda.monsefi@ukbonn.de (N.M.); ali.assayed@gmail.com (A.E.-S.A.); 2 Division of Cardiac Surgery, Heart Centre Siegburg, 53721 Siegburg, Germany; ali.bayram@helios-gesundheit.de (A.B.); abdul-sami.sirat@helios-gesundheit.de (S.S.); mirko.doss@helios-gesundheit.de (M.D.)

**Keywords:** coronary bypass surgery, off-pump bypass surgery, median sternotomy, minimally invasive, propensity matching

## Abstract

**Background/Objectives**: The minimally invasive approach through left mini-thoracotomy is a promising alternative to the median sternotomy for coronary artery bypass. The aim of this study was to compare the short-term outcomes of patients undergoing minimally invasive coronary artery bypass (MIDCAB) with off-pump coronary artery bypass through sternotomy (OPCAB) for single-vessel disease. **Methods**: From January 2017 to February 2023, 377 consecutive patients aged above 18 years undergoing off-pump bypass of the left anterior descending artery (LAD) with left internal thoracic artery underwent OPCAB. Propensity score matching was then applied. Primary endpoints were in-hospital mortality and 30-day mortality. **Results**: Prior to matching, 30-day mortality occurred in 2 (0.7%) patients in the MIDCAB group vs. 1 (1%) patient in the OPCAP group (*p* = 1). Transfusion of red blood cells (RBC) was required in 9.4% and 29% of patients within the MIDCAB and the OPCAB groups, respectively (*p* < 0.001). Median intensive care stay (ICU) was 1 [1–2] day in the MIDCAB group, vs. 2 [1–3] in the OPCAB (*p* < 0.001). In the matched cohort, 10% of MIDCAB patients received RBCs vs. 27.5% of OPCAB patients (*p* = 0.006). Median ICU stay was significantly lower in the MIDCAB group, 1 [1–2] vs. 2 [1–3] days. **Conclusions**: MIDCAB is as safe and effective as OPCAB for single coronary artery bypass of the LAD with the LITA in select patients. It is associated with a decreased ICU stay and lower transfusion rates when compared with OPCAB.

## 1. Introduction

The advantages of minimally invasive cardiac surgery (MICS) are well established in the current literature, and the current techniques and advancements in MICS are nowadays applied in a wide spectrum of cardiac interventions, varying from coronary revascularization, valvular disease, arrhythmia, and congenital malformations, to rare anatomical variations [1,2,3,4,5,6].

Less invasive techniques in off-pump bypass surgeries have been in continuous development during the past decades, since the first documented beating heart coronary anastomosis described by Kolessov in 1967 [7]. Minimally invasive direct coronary artery bypass (MIDCAB) was then first reported in the 1990s by Calafiore and Subramanian, offering the advantage of sparing the patient the trauma of a median sternotomy performed during off-pump coronary artery bypass surgery (OPCAB), by accessing the thoracic cavity through a small left anterior mini-thoracotomy (LAMT) [8,9]. With the convergence of outcomes between percutaneous coronary intervention and CABG in select patient populations, there has been a recent increase in attention regarding minimizing the surgical morbidity of coronary bypass operations [10].

The aim of this study was to present our experience with both MIDCAB and OPCAB and to compare the short-term outcomes of both procedures at two cardiac surgery referral centers in Germany.

## 2. Materials and Methods

The study was approved by the respective institutional review boards of both centers and individual patient consent was waived.

### 2.1. Study Design

We retrospectively enrolled 377 consecutive patients undergoing single-vessel off-pump coronary artery bypass surgery of the left anterior descending artery (LAD) with the left internal thoracic artery (LITA) between 2017 and 2023 in two high-volume centers in Germany (Heart Center Siegburg and Bonn University Hospital). All perioperative data were prospectively collected and electronically filed and catalogued in the electronic record database during the patients’ hospital stay. The choice of a minimally invasive access rather than a median sternotomy was based on an individual assessment of each patient’s characteristics, associated comorbidities, and anatomical conditions. The patient’s informed preference and the surgeon’s experience were also considered. Patients were then divided into a MIDCAB group and an OPCAB group. The pre-operative baseline characteristics of the patients between the two groups were then investigated and analyzed.

### 2.2. Surgical Methods

The surgical technique for MIDCAB surgery has previously been published [10]. All patients were placed in a supine position with a 30° elevation of the left thorax. After double-lumen intubation and induction of general anesthesia, a small 5–6 cm incision was made above or below the left nipple. The 4th or 5th intercostal space was then used to access the left pleural space and the left lung was deflated. Using a lifting retractor (Geister, Tuttlingen, Germany), a LITA graft was harvested. Systemic heparinization was then initiated, followed by a pericardiotomy and identification of the LAD. Distal anastomosis was performed off-pump with the help of a vacuum-based tissue stabilizer (Octopus Evolution, Medtronic, Minneapolis, MN, USA) and an intra-coronary shunt (Medtronic). A traction suture with tourniquets was placed with 4/0 Prolene proximally around the LAD. Air or saline solution insufflation was used to obtain a bloodless surgical field. After completing the anastomosis, the bypass flow was measured intra-operatively. Protamin was then administered 1:1 to Heparin. After placing a left pleural drain in to the left pleura, the thoracotomy was closed.

OPCAB was performed using the conventional method [11].

Following the procedure, patients were transferred to the intensive care unit (ICU) until they were extubated, awake and oriented, and free of catecholamine support. Then followed their stay on the regular cardiosurgical floor, where the early postoperative care, including wound management, mobilization, and treatment of any postoperative complication, took place, after which the patients were either discharged home, directly to a postoperative rehabilitation center, or transferred back to their respective referring center or hospital for further longer-term management.

### 2.3. Variables

The primary outcome was operation-related mortality, defined as in-hospital mortality occurring within 30 post-operative days. Other outcomes included in-hospital mortality of any cause, operation-related morbidity, ICU stay, post-operative in-hospital stay, need for red blood cell (RBC) transfusion, the number of transfused RBC units, and the patient destination after hospital discharge.

Defined as operation-related morbidity were postoperative myocardial infarction (MI), cerebrovascular events, intra-thoracic bleeding and bleeding requiring re-thoracotomy, low cardiac output syndrome, new-onset atrial fibrillation (AF), acute kidney injury and need for dialysis, pacemaker therapy, pneumothorax, need for extra-corporeal membrane oxygenation (ECMO), postoperative respiratory insufficiency that included postoperative prolonged intubation and need for reintubation, pneumonia, and wound revision surgery.

### 2.4. Statistical Analysis

Continuous variables were represented as mean ± standard deviation (SD) or as median [interquartile range] (IQR) depending on whether their distribution was observed as normal. The latter was visually inspected using a QQ plot and analyzed using the Shapiro–Wilk normality test. The IQR was expressed as an interval. Categorical data were reported as absolute counts *n* (%).

**Figure 1:**
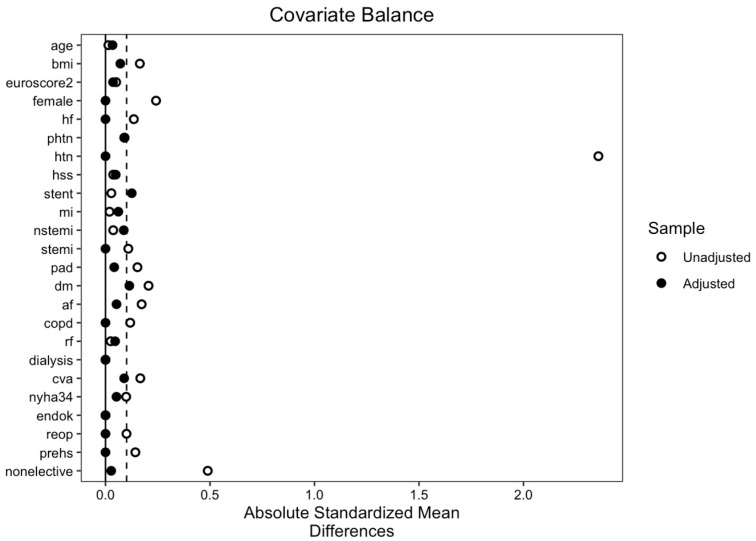
Absolute standardized mean differences of covariates. Abbreviations used: af, atrial fibrillation; bmi, body mass index; copd, chronic obstructive pulmonary disease; cva, cerebral vascular accident; dm, diabetes mellitus; endok, endocarditis; euroscore2. European system for cardiac operative risk evaluation score II; hf, heart failure; hss, left main coronary artery stenosis; htn, arterial hypertension; mi, myocardial infarction; nonelective, non-elective surgery; nstemi, non ST-elevation myocardial infarction; nyha34, New York Heart Association classification of III or IV; pad, peripheral arterial disease; phtn, pulmonary hypertension; prehs, history of previous cardiac surgery; reop, repeat procedure; rf, renal failure; stemi, ST-elevation myocardial infarction; stent, previous coronary stenting.

Propensity score matching was performed, in order to alleviate the selection bias innate to retrospective studies due to possible confounding factors. The propensity score for each patient was calculated using logistic regression, with adjustment for 24 key baseline variables (see Table 1 and Table 2). Matching was carried out using 1-to-1 nearest neighbor propensity score matching without replacement, with a caliper of 0.2. After matching, the absolute standardized mean difference (SMD) was used to assess the balance between the two groups, with an acceptable difference in means being less than 0.1. A Love plot showing the distribution of the SMD of each covariate prior to and following propensity matching is shown in Figure 1.

Comparison of continuous outcome variables prior to matching was performed with the non-parametric Mann–Whitney test, while categorical variables were compared using Pearson’s $\chi^{2}$ test and Fisher’s exact test where appropriate, depending on whether the minimum expected assumption was met. Comparisons between the two matched groups were carried out using either a paired *t*-test or a Wilcox signed rank sum test for continuous variables, depending on the normality of the distribution, while categorical variables were compared using McNemar’s mid-p test. In all cases, a two-tailed *p*-value of less than 0.05 was considered to indicate a statistically significant difference.

All statistical analyses were performed using R statistical software 4.3.2 (R Foundation for Statistical Computing, Vienna, Austria). The MatchIt package 4.5.5 was used for propensity score matching [12].

## 3. Results

### 3.1. Patient Characteristics

Between January 2017 and February 2023, a total of 377 patients underwent a single-vessel off-pump coronary artery bypass surgery of the LAD with the LITA. MIDCAB was performed in 277 (73.5%) patients, while 100 (26.5%) cases were performed via OPCAB. Across both groups, 100 (26.5%) patients were female, mean age was 64.9 ± 10.2 in the MIDCAB group and 64.8 ± 10 in the OPCAB group, and the median BMI was 27.4 [24.7–30.10] kg/m^2^ within the MIDCAB group and 27.1 [25.1–28.8] kg/m^2^ for the OPCAB group. Fifty-one (13.5%) cases were non elective and 61.5% of patients had a New York Heart Association Classification (NYHA) III/IV. The baseline characteristics of both groups are summarized in Table 1.

Matching resulted in two groups of 80 patients each, where the female sex represented 32.5% of each group, mean age was 65 ± 11 years in the MIDCAB group and 65 ± 9 years in the OPCAB group, the mean BMI was 26.8 [24.2–29] kg/m^2^ in the MIDCAB group and 27.1 [25.1–28.7] kg/m^2^ in the OPCAB group, while the number of patients receiving non-elective surgery was 25 (15.6%) overall, 15% of the MIDCAB group, and 16.2% of the OPCAB group, respectively. A total of 100 (62.5%) patients had a NYHA of III/IV across both groups, 41 (61.3%) for MIDCAB and 51 (62.7%) for OPCAB, respectively. The baseline characteristics of both groups after the 1:1 propensity matching are summarized in Table 2.

### 3.2. Outcomes

#### 3.2.1. Outcomes of Unmatched Groups

Post-operative outcomes across both unmatched groups are summarized in Table 3. Thirty-day mortality occurred in 2 (0.7%) patients in the MIDCAB group, which was similar in the OPCAB group with 1 (1%) case (*p*-value = 1). There was also no statistically significant difference in the incidence of in-hospital mortality, which occurred in 1.1% of the MIDCAB group vs. 1% of the OPCAB group (*p*-value = 1). Operative success was achieved in all patients, and no conversion to sternotomy within the MIDCAB group occurred.

MIDCAB was associated with significantly shorter ICU and total postoperative hospital stays however. Median ICU stay in the MIDCAB group was 1 [1–2] day vs. 2 [1–3] days in the OPCAB group (*p*-value < 0.001). Additionally, patients in the MIDCAB group had a median hospital stay of 6 [5–7] days, which was also significantly shorter than in the OPCAB group, with 7 [6–9] days (*p*-value < 0.001). Blood product transfusion requirements were also lower amongst the patients undergoing minimally invasive surgery, where RBC transfusion was required in only 9.4% of patients of the MIDCAB group, while 29% of patients operated on via sternotomy did indeed require packed red blood cell transfusion, *p*-value < 0.001. The median of transfused packed RBC units across both groups also showed a statistically significant difference, with 0 [0–0] vs. 0 [0–1] units, respectively (*p* < 0.001).

Regarding post-operative in-hospital morbidity, there was no significant differences between the MIDCAB group and the OPCAB group. While the requirement for PM implantation for arrhythmias was lower in the MIDCAB group, the difference was not statistically significant (0% vs. 2%, respectively, *p*-value = 0.07). The incidence of postoperative cardiovascular events, intrathoracic bleeding, kidney injury and dialysis, and respiratory insufficiency were similar across both groups.

#### 3.2.2. Outcomes of Matched Groups

Post-operative outcomes after propensity matching across both groups are summarized in Table 4.

Following propensity matching, the only remaining statistically significant differences between the two groups concerned ICU stay duration and the proportion of patients requiring packed RBC transfusion. Median ICU stay in the MIDCAB group was still shorter than in the sternotomy group (1 [1–2] vs. 2 [1–3] days, respectively, *p*-value < 0.001), and 8 (10%) patients from the lateral thoracotomy group required at least one unit of packed RBC transfusion vs. 22 (27.5%) from the sternotomy group (*p*-value = 0.006). There were no other significant differences between the two groups regarding other post-operative outcomes post matching.

In-hospital mortality and mortality within 30 days post-operatively, occurred in 2 (2.4%) MIDCAB cases, which was not significantly higher than in the OPCAB group with 2 (2.4%) cases (*p*-value = 1).

## 4. Discussion

The minimally invasive approach to coronary bypass surgery through mini-thoracotomy has been associated with fewer transfusions, shorter length of stay, and lower hospital cost, as well as a superior resource utilization profile that improves patient care and lowers cost when compared to approaches through sternotomy [7,8,9,13,14,15].

Aside from the better cosmesis, the smaller bone-sparing incision of the left mini-thoracotomy during MIDCAB has also been proposed to have a favorable effect on post-operative bleeding and blood product requirements [16,17,18]. Blood transfusions have previously been linked to an increased risk of postoperative morbidity and mortality in patients undergoing heart surgeries, including a longer hospital stay and increased costs [19]. Our results are indeed in accordance with the findings of the current literature, showing that fewer patients needed intraoperative and postoperative transfusions of red blood cells. Furthermore, our findings showed shorter intensive care unit stay durations for patients undergoing MIDCAB compared to sternotomy. Prolonged ICU stay has indeed been associated with higher morbidity and long-term mortality following patient discharge [20]. A shorter ICU-stay duration enables an earlier mobilization of patients and a faster recovery, while helping in preventing ICU-acquired weakness, which includes weakness, neuropathy, myopathy, as well as muscle atrophy [21].

However, concerns about minimal invasive coronary bypass through mini-thoracotomy have been raised, regarding an increased rate of incomplete revascularization, graft occlusion, and repeat revascularization when compared to OPCAB through sternotomy, while being similar in terms of early and mid-term mortality [22]. Although our results did indeed show similar early 30-day mortality and in-hospital mortality between the two techniques, surgical success was achieved in both groups. Graft patency was observed in all patients and post-operative myocardial infarction was absent in both groups.

Some of those concerns, however, were raised at an early stage of the adoption of MIDCAB, underscoring the importance of the use of adjunct techniques and technologies such as a cardiac tissue stabilizer or the use of a hybrid revascularization approach in combination with percutaneous coronary intervention [23,24]. Additionally, minimally invasive cardiac surgeries reportedly present a steep learning curve and additional technical challenges, influencing operative times and postoperative morbidity [25]. These technical challenges and concerns about the quality of coronary anastomoses were some of the difficulties described in earlier reports about MIDCAB [23]. This apparent disparity with our results can then be explained by the maturity of and the recent advances in minimally invasive techniques in cardiac surgeries at our centers [5,6,10,26,27,28,29,30,31,32]. Although MIDCAB, and other minimally invasive cardiac surgeries, are still relatively uncommon and mainly limited to specialist centers, they have been shown to be a very promising advancement in the field of cardiac surgery, being successfully and safely applied in a broad spectrum of surgical cardiac interventions [1]. When performed by experienced surgeons in minimally invasive approaches to cardiac surgery, MIDCAB should be considered as a first-line therapy for single-vessel bypass. MIDCAB is now the standard approach for LITA-LAD revascularization therapy at our institutions and currently remains under constant improvement, as we introduce the use of endoscopy as well as MIDCAB for multiple-vessel revascularization surgical therapy into our routine experience.

### Limitations

As discussed above, this study has an inherent selection bias due to the fact that it is a retrospective study and thus non-randomized. Although propensity score matching was performed to mitigate some of the treatment selection bias, the relatively small sample limited the statistical power of our analyses. A larger prospective randomized trial is needed in the future. Additionally, our study lacked long-term follow-up results and was limited to single-bypass surgery.

Furthermore, aspects of postoperative pain and pain management were not included in this retrospective study, as they were not systematically recorded for all patients included therein.

Finally, MIDCAB is known to have a steep learning curve; however, the surgeons operating on the patients of this study were experienced in the various techniques of minimally invasive cardiac surgery, which might have contributed to the favorable results.

## 5. Conclusions

In conclusion, MIDCAB is feasible, safe, and effective. This study showed that the endoscopic minimally invasive access for single-vessel bypass surgery was associated with a shorter ICU stay and lower blood product requirements compared to the OPCAB approach, while having a similar post-operative morbidity and mortality.

## Figures and Tables

**Table 1:** Patient characteristics of unmatched groups.

Variable	Overall (*n* = 377)	MIDCAB (*n* = 277)	OPCAB (*n* = 100)	SMD
Age * (mean ± SD)	65 ± 10	65 ± 10	65 ± 10	0.014
BMI * (median [IQR])	27.3 [24.8–29.6]	27.4 [24.7–30.1]	27.1 [25.1–28.8]	0.145
Euroscore II * (median [IQR])	1.8 [1.1–2.6]	1.8 [1.1–2.6]	1.9 [1.3–2.9]	0.049
Female *	100 (26.5%)	65 (23.5%)	35 (35%)	0.256
EF Range				0.322
>50	315 (83.6%)	227 (81.9%)	88 (88%)	
0–20	2 (0.5%)	2 (0.7%)	0 (0%)	
21–30	11 (2.9%)	6 (2.2%)	5 (5%)	
31–50	49 (13.0%)	42 (15.2%)	7 (7%)	
Heart Failure *	78 (20.7%)	53 (19.1%)	25 (25%)	0.142
Pulmonary HTN *	4 (1.1%)	2 (0.7%)	2 (2%)	0.11
HTN *	99 (26.3%)	97 (35.0%)	2 (2%)	0.939
LMA stenosis *	29 (7.7%)	22 (7.9%)	7 (7%)	0.035
Previous PCI	110 (29.2%)	74 (26.7%)	36 (36%)	0.201
Previous Stenting *	3 (0.8%)	2 (0.7%)	1 (1%)	0.03
MI *	77 (20.4%)	56 (20.2%)	21 (21%)	0.019
NSTEMI *	31 (8.2%)	22 (7.9%)	9 (9%)	0.038
STEMI *	21 (5.6%)	17 (6.1%)	4 (4%)	0.098
PAD *	25 (6.6%)	15 (5.4%)	10 (10%)	0.173
DM *	73 (19.4%)	47 (17.0%)	26 (26%)	0.221
AF *	34 (9.0%)	28 (10.1%)	6 (6%)	0.151
COPD *	38 (10.1%)	25 (9.0%)	13 (13%)	0.127
Renal failure *	32 (8.5%)	24 (8.7%)	8 (8%)	0.024
Dialysis *	0 (0%)	0 (0%)	0 (0%)	<0.001
History of stroke *	14 (3.7%	12 (4.3%)	2 (2%)	0.133
NYHA				0.378
I	25 (6.6%)	16 (5.8%)	9 (9%)	
II	120 (31.8%)	94 (33.9%)	26 (26%)	
III	216 (57.3%)	161 (58.1%)	55 (55%)	
IV	16 (4.2%)	6 (2.2%)	10 (10%)	
NYHA III–IV *	232 (61.5%)	167 (60.3%)	65 (65%)	0.098
Endocarditis *	0 (0%)	0 (0%)	0 (0%)	<0.001
Reoperation *	1 (0.3%)	0 (0%)	1 (1%)	0.142
Previous heart surgery *	2 (0.5%)	0 (0%)	2 (2%)	0.202
Non elective *	51 (13.5%)	21 (7.6%)	30 (30%)	0.599

All values are represented as absolute number *n* (%) unless specified otherwise. * Variables used in propensity score matching. Abbreviations: AF: atrial fibrillation, COPD: chronic obstructive pulmonary disease, DM: diabetes mellitus, EF: ejection fraction, HTN: hypertension, IQR: interquartile range, LMA: left main artery, MI: myocardial infarct, NSTEMI: non ST elevation myocardial infarct, NYHA: New York Heart Association, PAD: peripheral artery disease, PCI: percutaneous coronary intervention, SD: standard deviation, STEMI: ST elevation myocardial infarct.

**Table 2:** Patient characteristics of matched groups.

Variable	Overall (*n* = 160)	MIDCAB (*n* = 80)	OPCAB (*n* = 80)	SMD
Age * (mean ± SD)	65 ± 10	65 ± 11	65 ± 9	0.033
BMI * (median [IQR])	27 [25–29]	27 [24–29]	27 [25–29]	0.063
Euroscore II * (median [IQR])	1.7 [1.1–2.6]	1.5 [1–2.9]	1.8 [1.2–2.6]	0.047
Female *	52 (32.5%)	26 (32.5%)	26 (32.5%)	<0.001
EF Range				0.405
>50	136 (85%)	63 (78.8%)	73 (91.2%)	
21–30	6 (3.8%)	3 (3.8%)	3 (3.8%)	
31–50	18 (11.2%)	14 (17.5%)	4 (5.0%)	
Heart Failure *	34 (21.2%)	17 (21.2%)	17 (21.2%)	<0.001
Pulmonary HTN *	1 (0.6%)	1 (1.2%)	0 (0%)	0.159
HTN *	2 (1.2%)	1 (1.2%)	1 (1.2%)	<0.001
LMA stenosis *	11 (6.9%)	6 (7.5%)	5 (6.2%)	0.049
Previous PCI	39 (24.4%)	14 (17.5%)	25 (31.2%)	0.324
Previous Stenting *	3 (1.9%)	2 (2.5%)	1 (1.2%)	0.092
MI *	30 (18.8%)	14 (17.5%)	16 (20.0%)	0.064
NSTEMI *	16 (10%)	9 (11.2%)	7 (8.8%)	0.083
STEMI *	8 (5%)	4 (5.0%)	4 (5.0%)	<0.001
PAD *	15 (9.4%)	8 (10.0%)	7 (8.8%)	0.043
DM *	36 (22.5%)	16 (20.0%)	20 (25.0%)	0.12
AF *	7 (4.4%)	3 (3.8%)	4 (5.0%)	0.061
COPD *	18 (11.2%)	9 (11.2%)	9 (11.2%)	<0.001
Renal failure *	15 (9.4%)	7 (8.8%)	8 (10%)	0.043
Dialysis *	0 (0%)	0 (0%)	0 (0%)	<0.001
History of stroke *	5 (3.1%)	3 (3.8%)	2 (2.5%)	0.072
NYHA				0.278
I	12 (7.5%)	5 (6.2%)	7 (8.8%)	
II	48 (30%)	26 (32.5%)	22 (27.5%)	
III	89 (55.6%)	46 (57.5%)	43 (53.8%)	
IV	11 (6.9%)	3 (3.8%)	8 (10%)	
NYHA III–IV *	100 (62.5%)	49 (61.3%)	51 (63.7%)	0.052
Endocarditis *	0 (0%)	0 (0%)	0 (0%)	<0.001
Reoperation *	0 (0%)	0 (0%)	0 (0%)	<0.001
Previous heart surgery *	0 (0%)	0 (0%)	0 (0%)	<0.001
Non elective *	25 (15.6%)	12 (15%)	13 (16.2%)	0.034

All values are represented as absolute number *n* (%) unless specified otherwise. * Variables used in propensity score matching. Abbreviations: AF: atrial fibrillation, COPD: chronic obstructive pulmonary disease, DM: diabetes mellitus, EF: ejection fraction, HTN: hypertension, IQR: interquartile range, LMA: left main artery, MI: myocardial infarct, NSTEMI: non-ST elevation myocardial infarct, NYHA: New York Heart Association, PAD: peripheral artery disease, PCI: percutaneous coronary intervention, SD: standard deviation, STEMI: ST elevation myocardial infarct.

**Table 3:** Outcomes of unmatched groups.

Outcome	MIDCAB (*n* = 277)	OPCAB (*n* = 100)	*p*-Value
ICU (days) (median [IQR])	1 [1–2]	2 [1–3]	<0.001
Stay (days) (median [IQR])	6 [5–7]	7 [6–9]	<0.001
pRBC (units) (median [IQR])	0 [0–0]	0 [0–1]	<0.001
CV events	8 (2.9%)	2 (2%)	1
Stroke	0 (0%)	0 (0%)	NA
Delirium	8 (2.9%)	2 (2%)	1
Delirium requiring Rx	3 (1.1%)	2 (2%)	0.612
Delirium–temporary	5 (1.8%)	0 (0%)	0.331
Intrathoracic bleed	1 (0.4%)	2 (2%)	0.173
Intrathoracic bleed requiring rethoracotomy	1 (0.4%)	1 (1%)	0.461
Required transfusion	26 (9.4%)	29 (29%)	<0.001
LCOS	2 (0.7%)	3 (3%)	0.119
AKI	10 (3.6%)	8 (8%)	0.099
Dialysis	3 (1.1%)	0 (0%)	0.569
AF	8 (2.9%)	3 (3%)	1
Arrythmia requiring PM	0 (0%)	2 (2%)	0.07
Pneumothorax	3 (1.1%)	0 (0%)	0.569
Pneumothorax requiring Rx	1 (0.4%)	0 (0%)	1
ECMO	4 (1.4%)	0 (0%)	0.577
Respiratory insufficiency	7 (2.5%)	4 (4%)	0.492
Respiratory insufficiency requiring invasive Rx	5 (1.8%)	3 (3%)	0.442
Pneumonia	5 (1.8%)	5 (5%)	0.139
Wound revision	0 (0%)	0 (0%)	NA
Discharged home	175 (63.2%)	57 (57%)	0.276
Discharged to rehabilitation	63 (22.7%)	26 (26%)	0.511
Discharged to another hospital	37 (13.4%)	16 (16%)	0.515
30-day mortality	2 (0.7%)	1 (1%)	1
In-hospital mortality	3 (1.1%)	1 (1%)	1

All values are represented as absolute number *n* (%) unless specified otherwise. Abbreviations: AF: atrial fibrillation, AKI: acute kidney injury, CV: cerebrovascular, ECMO: extracorporeal membrane oxygenation, ICU: intensive care unit, IQR: interquartile range, LCOS: low cardiac output syndrome, MI: myocardial infarct, NA: not applicable, PM: pacemaker, pRBC: packed red blood cells, Rx: treatment.

**Table 4:** Outcomes of matched groups.

Outcome	MIDCAB (*n* = 80)	OPCAB (*n* = 80)	*p*-Value
ICU (days) (median [IQR])	1 [1–2]	2 [1–3]	<0.001
Stay (days) (median [IQR])	7 [5–7]	7 [6–8.25]	0.077
pRBC (units) (median [IQR])	0 [0–0]	0 [0–1]	0.088
CV events	1 (1.2%)	2 (2.5%)	0.625
Stroke	0 (0%)	0 (0%)	1
Delirium	1 (1.2%)	2 (2.5%)	0.625
Delirium requiring Rx	0 (0%)	2 (2.5%)	0.25
Delirium–temporary	1 (1.2%)	0 (0%)	0.5
Intrathoracic bleed	0 (0%)	1 (1.2%)	0.5
Intrathoracic bleed requiring rethoracotomy	1 (1.2%)	0 (0%)	0.5
Required transfusion	8 (10%)	22 (27.5%)	0.006
LCOS	1 (1.2%)	3 (3.8%)	0.375
AKI	5 (6.2%)	7 (8.8%)	0.581
Dialysis	1 (1.2%)	0 (0%)	0.5
AF	0 (0%)	3 (3.8%)	0.125
Arrythmia requiring PM	0 (0%)	2 (2.5%)	0.25
Pneumothorax	1 (1.2%)	0 (0%)	0.5
Pneumothorax requiring Rx	0 (0%)	0 (0%)	1
ECMO	2 (2.5%)	0 (0%)	0.25
Respiratory insufficiency	1 (1.2%)	4 (5%)	0.22
Respiratory insufficiency requiring invasive Rx	1 (1.2%)	3 (3.8%)	0.375
Pneumonia	1 (1.2%)	4 (5%)	0.219
Wound revision	0 (0%)	0 (0%)	1
Discharged home	54 (67.5%)	47 (58.8%)	0.25
Discharged to rehabilitation	21 (26.2%)	20 (25%)	0.85
Discharged to another hospital	5 (6.2%)	12 (15%)	0.07
30-day mortality	1 (1.2%)	1 (1.2%)	1
In-hospital mortality	1 (1.2%)	1 (1.2%)	1

All values are represented as absolute number *n* (%) unless specified otherwise. Abbreviations: AF: atrial fibrillation, AKI: acute kidney injury, CV: cerebrovascular, ECMO: extracorporeal membrane oxygenation, ICU: intensive care unit, IQR: interquartile range, LCOS: low cardiac output syndrome, MI: myocardial infarct, PM: pacemaker, pRBC: packed red blood cells, Rx: treatment.

## Data Availability

The data presented in this study are available on request from the corresponding author.
